# Supplementary material for: A fault-intrusion-tolerant system and deadline-aware algorithm for scheduling scientific workflow in the cloud
Source: PeerJ Comput Sci. 2021 Nov 2;7:e747. doi: 10.7717/peerj-cs.747 (PMC8576554; doi:10.7717/peerj-cs.747)
Supplement: Supplemental Information 6 [file peerj-cs-07-747-s006.zip › Myproject1.1/docs/org/cloudbus/cloudsim/distributions/class-use/ContinuousDistribution.html]

Uses of Interface org.cloudbus.cloudsim.distributions.ContinuousDistribution


JavaScript is disabled on your browser.


- Overview
- Package
- Class
- Use
- Tree
- Deprecated
- Index
- Help

- Prev
- Next

- Frames
- No Frames

- All Classes

## Uses of Interface org.cloudbus.cloudsim.distributions.ContinuousDistribution

- Packages that use ContinuousDistribution

  | Package | Description |
  | --- | --- |
  |  |  |
  | --- | --- |
  | org.cloudbus.cloudsim |  |
  | org.cloudbus.cloudsim.distributions |  |
- - ### Uses of ContinuousDistribution in org.cloudbus.cloudsim

    Methods in org.cloudbus.cloudsim with parameters of type ContinuousDistribution

    | Modifier and Type | Method and Description |
    | --- | --- |
    |  |  |
    | --- | --- |
    | `boolean` | HarddriveStorage.`setAvgSeekTime(double seekTime, ContinuousDistribution gen)` Sets the average seek time and a new generator of seek times in seconds. |
  - ### Uses of ContinuousDistribution in org.cloudbus.cloudsim.distributions

    Classes in org.cloudbus.cloudsim.distributions that implement ContinuousDistribution

    | Modifier and Type | Class and Description |
    | --- | --- |
    |  |  |
    | --- | --- |
    | `class` | `ExponentialDistr` An exponential number generator. |
    | `class` | `GammaDistr` The Class GammaDistr. |
    | `class` | `LognormalDistr` The Class LognormalDistr. |
    | `class` | `LomaxDistribution` The Class LomaxDistribution. |
    | `class` | `ParetoDistr` The Class ParetoDistr. |
    | `class` | `UniformDistr` A random number generator based on the Uniform distribution. |
    | `class` | `WeibullDistr` The Class WeibullDistr. |
    | `class` | `ZipfDistr` The Class ZipfDistr. |

- Overview
- Package
- Class
- Use
- Tree
- Deprecated
- Index
- Help

- Prev
- Next

- Frames
- No Frames

- All Classes
